# Epidemiology and Transmission Dynamics of West Nile Virus Disease

**DOI:** 10.3201/eid1108.050289a

**Published:** 2005-08

**Authors:** Edward B. Hayes, Nicholas Komar, Roger S. Nasci, Susan P. Montgomery, Daniel R. O'Leary, Grant L. Campbell

**Affiliations:** * Centers for Disease Control and Prevention, Fort Collins, Colorado, USA

**Keywords:** West Nile virus, encephalitis, prevention, zoonosis, ecology, mosquito control, blood transfusion, organ tranplantation, pediatrics, intrauterine

## Abstract

From 1937 until 1999, West Nile virus (WNV) garnered scant medical attention as the cause of febrile illness and sporadic encephalitis in parts of Africa, Asia, and Europe. After the surprising detection of WNV in New York City in 1999, the virus has spread dramatically westward across the United States, southward into Central America and the Caribbean, and northward into Canada, resulting in the largest epidemics of neuroinvasive WNV disease ever reported. From 1999 to 2004, >7,000 neuroinvasive WNV disease cases were reported in the United States. In 2002, WNV transmission through blood transfusion and organ transplantation was described for the first time, intrauterine transmission was first documented, and possible transmission through breastfeeding was reported. This review highlights new information regarding the epidemiology and dynamics of WNV transmission, providing a new platform for further research into preventing and controlling WNV disease.

## Abstract 2

Since 1999, >16,000 cases in the United Stateswere transmitted by *Culex* mosquitoes.

West Nile virus (WNV) was first detected in the Western Hemisphere in 1999 during an outbreak of encephalitis in New York City. Over the next 5 years, the virus spread across the continental United States as well as north into Canada, and southward into the Caribbean Islands and Latin America (1). This article highlights new information about the epidemiology and transmission dynamics of human WNV disease obtained over the past 5 years of intensified research.

## Epidemiology

WNV is transmitted primarily by the bite of infected mosquitoes that acquire the virus by feeding on infected birds. The intensity of transmission to humans is dependent on abundance and feeding patterns of infected mosquitoes and on local ecology and behavior that influence human exposure to mosquitoes. Although up to 55% of affected populations became infected during epidemics in Africa, more recent outbreaks in Europe and North America have yielded much lower attack rates (1,2). In the area of most intense WNV transmission in Queens, New York, in 1999, ≈2.6% of residents were infected (most of these were asymptomatic infections), and similarly low prevalence of infection has been seen in other areas of the United States (3,4). WNV outbreaks in Europe and the Middle East since 1995 appear to have caused infection in <5% of affected populations (1,5). These levels of infection are too low to decrease the frequency of epidemics or modulate their intensity through protective immunity.

Data on the incidence of WNV in most of the world are not readily available. WNV transmission has been reported in Europe, the Middle East, Africa, India, parts of Asia, Australia (in the form of Kunjin virus, a subtype of WNV), North America, and parts of Central America and the Caribbean (1,6). In recent years human WNV disease in the Eastern Hemisphere has been reported mostly from areas in the Mediterranean Basin: in Algeria in 1994, Morocco in 1996, Tunisia in 1997 and 2003, Romania in 1996 through 2000, the Czech Republic in 1997, Israel in 1999 and 2000, Russia in 1999 through 2001, and France in 2003 (1,6,7). Enzootics involving horses were reported in Morocco in 1996 and 2003, Italy in 1998, Israel in 2000, and southern France in 2000, 2003, and 2004 (6–8).

**Figure 1:**
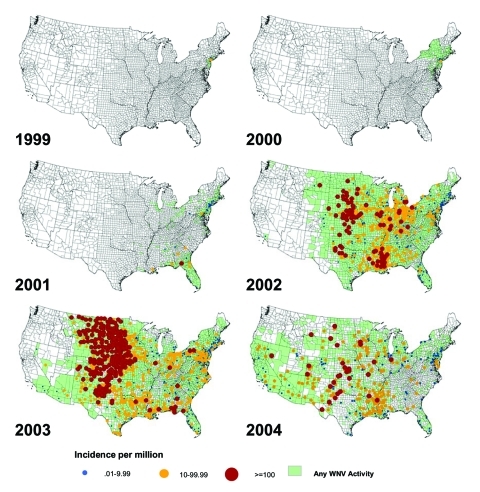
Reported incidence of neuroinvasive West Nile virus disease by county, United States, 1999–2004. Reported to Centers for Disease Control and Prevention by states through April 21, 2005.

In the Western Hemisphere, most human WNV disease has occurred in the United States. Since the virus was detected in New York from 1999 through 2004, 16,706 cases have been reported to the Centers for Disease Control and Prevention (CDC); 7,096 of these were classified as neuroinvasive disease, 9,268 as West Nile fever (WNF), and 342 had other or unspecified clinical presentation (reported through June 8, 2005; the proportion of total cases reported that are neuroinvasive disease is artificially higher than what is believed to occur naturally since neuroinvasive disease is more likely to be reported than WNF or asymptomatic infection) (Table 1). Transmission of WNV has spread dramatically from New York to the north, south, and west (Figure 1). From 2002 to 2003, the most intense transmission shifted from the Midwest and south-central states to the western plains and Front Range of the Rocky Mountains. In 2004, most WNV disease cases were reported in California, Arizona, and western Colorado, but foci of highest incidence were scattered across the United States (Figure 1). In the East, WNV transmission recurred for 6 consecutive years with the highest number of human disease cases reported in 2003, indicating that WNV disease has become seasonally endemic. In Canada, transmission of WNV to humans has been documented in Quebec, Ontario, Manitoba, Saskatchewan, and Alberta, and WNV-infected birds have also been found in New Brunswick and Nova Scotia (http://www.phac-aspc.gc.ca/wnv-vwn). Evidence of WNV transmission has been reported from the Cayman Islands, Jamaica, Dominican Republic, Mexico, Guadeloupe, El Salvador, Belize, Puerto Rico, and Cuba, but only 1 human case has been reported from Mexico and 1 from the Cayman Islands (http://www.paho.org/English/DD/PIN/ptoday15_oct03.htm; http://www.paho.org/English/AD/DPC/CD/wnv.htm; http://www.cenave.gob.mx/von/default.asp; http://www.serc.si.edu/labs/avian/wnv.jsp) (1). The paucity of human cases thus far in Latin America and the Caribbean is surprising, considering the ecologic conditions that favor arbovirus transmission in these areas. WNV isolated from a bird in Mexico in 2003 appeared to be attenuated, but whether viral mutation accounts for the scarcity of human disease remains to be seen (9).

**Table 1:** Human West Nile virus disease cases by clinical syndrome, United States, 1999–2004*

Year	Total cases	Neuroinvasive cases	West Nile fever cases	Other clinical /unspecified	Deaths
1999	62	59	3	0	7
2000	21	19	2	0	2
2001	66	64	2	0	9
2002	4,156	2,946	1,162	48	284
2003	9,862	2,866	6,830	166	264
2004*	2,539	1,142	1,269	128	100
Total	16,706	7,096	9,268	342	666

*Reported to the Centers for Disease Control and Prevention as of June 8, 2005.

The incidence of WNV disease is seasonal in the temperate zones of North America, Europe, and the Mediterranean Basin, with peak activity from July through October (6,10). In the United States, the transmission season has lengthened as the virus has moved south; in 2003, onset of human illness began as late as December, and in 2004 as early as April (CDC, unpub. data). Transmission of WNV in southern Africa and of Kunjin virus in Australia increases in the early months of the year after heavy spring and summer rainfall (2,11).

**Figure 2:**
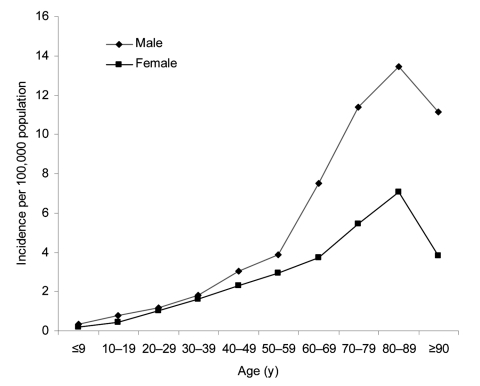
Reported incidence of neuroinvasive West Nile virus disease by age group and sex, United States, 1999–2004. Reported to the Centers for Disease Control and Prevention by states through April 14, 2005.

In the United States, persons of all ages appear to be equally susceptible to WNV infection, but the incidence of neuroinvasive WNV disease and death increases with age, especially among those 60 to 89 years of age, and is slightly higher among male patients (Figure 2) (10). During 2002, the median age among neuroinvasive disease cases was 64 years (range 1 month to 99 years), compared to a median age of 49 years (range 1–97 years) for WNF cases (10). Of the 2,942 neuroinvasive disease cases, 276 (9%) were fatal (10). Although severe disease occurs primarily in adults, neuroinvasive disease in children has been reported. From 2002 through 2004, 1,051 WNV disease cases among children <19 years of age were reported in the United States; 317 (30%) had neuroinvasive disease; and 106 (34%) of these were <10 years (CDC, unpub. data; reported through June 8, 2005). Two (0.6%) pediatric patients with neuroinvasive WNV disease died: an infant with underlying lissencephaly and a 14-year-old boy with immune dysfunction.

The most important risk factor for acquiring WNV infection is exposure to infected mosquitoes. In Romania the risk for WNV infection was higher among persons with mosquitoes in their homes and with flooded basements (12). An analysis of the locations of WNV disease cases during the 1999 outbreak in New York found that cases were clustered in an area with higher vegetation cover, indicating favorable mosquito habitat (13). A study of the outbreak in Chicago in 2002 indicated that human disease cases tended to occur in areas with more vegetation, older housing, lower population density, predominance of older Caucasian residents, and proximity to dead birds, but the effects of these variables were influenced by differences in mosquito abatement efforts (14). Risk factors for infection not related to mosquito exposure include receiving blood transfusions or organ donations, maternal infection during pregnancy or breastfeeding, and occupational exposure to the virus (15–17).

Apart from older age and immunosuppression after organ transplantation, the risk factors for the development of severe neuroinvasive WNV disease have yet to be determined (10,16). Underlying hypertension, cerebrovascular disease, and diabetes have been considered as possible predisposing factors; further study may elucidate the role of these or other host factors that might modify the risk for severe disease or death (12). Genetic predisposition for severe disease has been described in mice but has not yet been elucidated in humans (18). The role of innate and adaptive immune responses in determining outcome deserves further study.

## Nonmosquitoborne WNV Transmission

In 2002, intrauterine WNV transmission was documented for the first time (15). A 20-year-old woman had onset of WNV disease in week 27 of gestation. Her infant was born at term with chorioretinitis and cystic damage of cerebral tissue. Intensified surveillance identified 4 other mothers who had WNV illness during pregnancy, 3 of whom delivered infants with no evidence of WNV infection; all 3 infants appeared normal at birth and at 6 months of age (15). The fourth woman delivered prematurely; her infant had neonatal respiratory distress but was not tested for WNV infection. In 2003, CDC received reports of 74 women infected with WNV during pregnancy; most of these women followed up to date have delivered apparently healthy infants (CDC, unpub. data).

Probable WNV transmission through breast milk was also reported in 2002 (15). A 40-year-old woman acquired WNV infection from blood transfused shortly after she delivered a healthy infant. WNV nucleic acid was detected in her breast milk, and immunoglobulin (Ig) M antibody was found in her infant, who remained healthy. No other instances of possible WNV transmission through breast milk have been reported. Until more data are available, and because the benefits of breastfeeding are well documented, mothers should be encouraged to breastfeed even in areas of ongoing WNV transmission.

Transmission of WNV through blood transfusion was first documented during the 2002 WNV epidemic in North America (15). In June 2003, blood collection agencies in the United States and Canada enhanced donor deferral and began screening blood donations with experimental nucleic acid amplification tests. During 2003 and 2004, >1,000 potentially WNV-viremic blood donations were identified, and the corresponding blood components were sequestered. Nevertheless, 6 WNV cases due to transfusion were documented in 2003, and at least 1 was documented in 2004, indicating that infectious blood components with low concentrations of WNV may escape current screening tests (19). One instance of possible WNV transmission through dialysis has been reported (20).

WNV transmission through organ transplantation was also first described during the 2002 epidemic (15). Chronically immunosuppressed organ transplant patients appear to have an increased risk for severe WNV disease, even after mosquito-acquired infection (16). During 2002, the estimated risk of neuroinvasive WNV disease in solid organ transplant patients in Toronto, Canada, was approximately 40 times greater than in the general population (16). Whether other immunosuppressed or immunocompromised patients are at increased risk for severe WNV disease is uncertain, but severe WNV disease has been described among immunocompromised patients.

WNV infection has been occupationally acquired by laboratory workers through percutaneous inoculation and possibly through aerosol exposure (21,22). An outbreak of WNV disease among turkey handlers at a turkey farm raised the possibility of aerosol exposure (17).

## Dynamics of Transmission: Vectors

WNV is transmitted primarily by *Culex* mosquitoes, but other genera may also be vectors (23). In Europe and Africa, the principal vectors are *Cx*. *pipiens*, *Cx*. *univittatus*, and *Cx*. *antennatus*, and in India, species of the *Cx*. *vishnui* complex (6,24). In Australia, Kunjin virus is transmitted primarily by *Cx*. *annulirostris* (11). In North America, WNV has been found in 59 different mosquito species with diverse ecology and behavior; however, <10 of these are considered to be principal WNV vectors (CDC, unpub. data) (23,25,26). In 2001, 57% of the positive mosquito pools in the Northeast were *Cx*. *pipiens*, the northern house mosquito, a moderately efficient vector that feeds on birds and mammals (Table 2). In 2002, *Cx*. *pipiens* made up more than half of the WNV-positive pools, but *Cx*. *quinquefasciatus*, the southern house mosquito, generally considered a moderate- to low-efficiency vector, appeared to be the predominant vector in the South. *Cx*. *tarsalis*, 1 of the most efficient WNV vectors evaluated in laboratory studies, was the predominant vector west of the Mississippi River (CDC, unpub. data) (26).

**Table 2:** West Nile virus (WNV)–positive mosquito pools, by species, United States, 2001–2004*

2001		2002		2003		2004 (through 11/30/2004)	
Positive pools, n = 612†		Positive pools, n = 3,720†		Positive pools, n = 5,538†		Positive pools, n = 4,755†	
Species	% of pools	Species	% of pools	Species	% of pools	Species	% of pools
*Culex pipiens*	57.0	*Cx*. *pipiens*	47.0	*Cx*. *tarsalis*	31.5	*Cx*. *quinquefasciatus*	51.4
*Cx*. *restuans*	12.4	*Cx*. *quinquefasciatus*	19.1	*Cx*. *pipiens*	20.8	*Cx*. *tarsalis*	20.4
*Cx*. *salinarius*	11.4	*Cx*. *restuans*	9.1	*Cx*. *quinquefasciatus*	19.1	*Cx*. *pipiens*	12.7
*Culiseta melanura*	4.2	*Cx*. *tarsalis*	7.6	*Cx*. *restuans*	15.3	*Cx*. *restuans*	4.4
*Cx*. *quinquefasciatus*	2.1	*Cx*. *salinarius*	3.6	*Cx*. *salinarius*	4.5	*Cx*. *erythrothorax*	3.6
*Ochlerotatus*. *triseriatus*	2.1	*Aedes albopictus*	2.0	*Ae*. *vexans*	2.3		
21 other species‡	10.6	23 other species‡	11.5	35 other species‡	6.4	35 other species‡	7.5

*Data were derived from reports submitted by state health departments to the Centers for Disease Control and Prevention's Arbonet surveillance system. Mosquito specimens were collected, identified, and tested in the respective state surveillance systems. Pools were reported as positive if they contained detectable levels of one of the following: infectious WNV, WNV RNA, WN viral antigen. †Includes only WNV-positive pools reported as monospecific, i.e., excludes mixed pools (e.g., *Cx*. *pipiens*/*restuans*) or pools identified only to genus (e.g., *Culex* species). ‡No other species individually comprised ≥2.0% of the WNV-positive pools.

During 2003, as WNV activity progressed westward, *Cx*. *tarsalis* became the most commonly reported WNV-positive mosquito species, making up 32% of the positive pools reported, followed by *Cx*. *pipiens*, *Cx*. *quinquefasciatus*, and *Cx*. *restuans* (Table 2). *Cx*. *salinarius* and *Cx*. *nigripalpus* may be important vectors in areas where they are abundant (26). During 2004, when large epidemics occurred in the southwestern United States, the most commonly reported WNV-positive species was *Cx*. *quinquefasciatus*, which made up over half of the positive pools, followed by *Cx*. *tarsalis* and *Cx*. *pipiens* (Table 2).

The intensity of WNV transmission is determined primarily by the abundance of competent mosquitoes and the prevalence of infection in mosquitoes. The estimated prevalence of infection, measured as the minimum infection rate (MIR), that is needed to produce epidemics is uncertain. Toward the end of the 1999 New York epidemic, the WNV MIR for all *Culex* mosquitoes sampled in the area was 0.3% with MIRs of individual collections, ranging from 0.07% to 5.7% (27). During the 2000 Staten Island epidemic, the MIRs in mixed *Cx*. *pipiens*/*restuans* pools ranged from 0.5% to 1.6% and the MIR in *Cx*. *salinarius* from 0.3% to 1.2% (28). Relatively low MIRs in *Cx*. *restuans* (0.2%), *Cx*. *pipiens* (0.1%) and *Cx*. *salinarius* (0.1%) in Connecticut during 2000 were associated with an intense epizootic, but apparently a low risk for humans (29). In 2001, moderate to high MIRs in *Cx*. *quinquefasciatus* (0.5%) and *Cx*. *nigripalpus* (1.1%) were associated with epizootic and epidemic transmission in Florida (30). In some North American outbreaks, MIRs as high as 15% have been observed (CDC, unpub. data). Vertical transmission of WNV has been experimentally demonstrated in *Cx*. *pipiens*, *Cx*. *quinquefasciatus*, and *Cx*. *tarsalis*, and the virus has been isolated from hibernating female mosquitoes, which may provide a mechanism for persistence of the virus in colder latitudes through the winter and reemergence of transmission in the spring (31,32).

Although both soft and hard ticks can become infected with WNV, they are unlikely to play a substantial role in WNV transmission. In the laboratory, *Argas arboreus* ticks transmitted WNV to chickens, and *Ornithodoros savignyi*, *O*. *maritimus*, *O*. *erraticus*, and *O*. *moubata* transmitted WNV to mice (33). However, of the hard ticks *Amblyomma americanum*, *Ixodes scapularis*, *I*. *ricinus, Dermacentor variabilis*, and *D*. *andersoni*, the last 4 species became infected with WNV, but none transmitted the virus by subsequent bite (33,34).

## Dynamics of Transmission: Vertebrate Hosts

Laboratory studies have demonstrated that 74%–100% of *Cx*. *tarsalis* mosquitoes become infected after consuming blood meals with WNV concentrations of 10^7.1^ plaque-forming units (PFU)/mL, while only 0%–36% become infected after consuming a meal containing 10^4.9^ PFU/mL (35). The maximum estimated concentration of WNV in human blood tested during screening of blood donors in 2002 was approximately 10^3.2^ PFU/mL (S. Stramer, M. Busch, M. Strong, pers. comm.). Thus, it appears unlikely that humans exhibit WNV viremia levels of sufficient magnitude to infect mosquitoes.

Birds are presumed to be the most important amplifying hosts of WNV. In laboratory studies, species in the orders Passeriformes (song birds), Charadriiformes (shorebirds), Strigiformes (owls), and Falconiformes (hawks) developed viremia levels sufficient to infect most feeding mosquitoes, whereas species of Columbiformes (pigeons), Piciformes (woodpeckers), and Anseriformes (ducks) did not (23,36). Certain passerines, including common grackles (*Quiscalus quiscula*), various corvids (crows, jays, magpies), house finches (*Carpodacus mexicanus*), and house sparrows (*Passer domesticus*) were highly infectious to mosquitoes and had mortality rates >40%. Field studies during and after WNV outbreaks in several areas of the United States have confirmed that house sparrows were abundant and frequently infected with WNV, characteristics that would allow them to serve as important amplifying hosts (23,25,37). The importance of birds in dispersing WNV remains speculative. Local movements of resident, nonmigratory birds and long-range travel of migratory birds may both contribute to the spread of WNV (38,39).

Although WNV was isolated from rodents in Nigeria and a bat in India, most mammals do not appear to generate viremia levels of sufficient titer to contribute to transmission (24,40–42). Three reptilian and 1 amphibian species (red-ear slider, garter snake, green iguana, and North American bullfrog) were found to be incompetent as amplifying hosts of a North American WNV strain, and no signs of illness developed in these animals (43). Viremia levels of sufficient titer to infect mosquitoes were found after experimental infection of young alligators (*Alligator mississippiensis*) (44). In Russia, the lake frog (*Rana ridibunda*) appears to be a competent reservoir (45).

Nonmosquitoborne WNV transmission has been observed or strongly suspected among farmed alligators, domestic turkeys in Wisconsin, and domestic geese in Canada (17,46,47). Transmission through close contact has been confirmed in both birds and alligators in laboratory conditions but has yet to be documented in wild vertebrate populations (23,36,44).

## Control of WNV Transmission

Avoiding human exposure to WNV-infected mosquitoes remains the cornerstone for preventing WNV disease. Source reduction, application of larvicides, and targeted spraying of pesticides to kill adult mosquitoes can reduce the abundance of mosquitoes, but demonstrating their impact on the incidence of human WNV disease is challenging because of the difficulty in accounting for all determinants of mosquito abundance and human exposure. One study indicated that clustering of human WNV disease in Chicago varied between mosquito abatement districts, suggesting that mosquito control may have some impact on transmission to humans (14).

Persons in WNV-endemic areas should wear insect repellent on skin and clothes when exposed to mosquitoes and avoid being outdoors during dusk to dawn when mosquito vectors of WNV are abundant. Of insect repellents recommended for use on skin, those containing *N*,*N*-diethyl-m-toluamide (DEET), picaridin (KBR-3023), or oil of lemon eucalyptus (*p*-menthane-3,8 diol) provide long-lasting protection (48). Both DEET and permethrin provide effective protection against mosquitoes when applied to clothing. Persons' willingness to use DEET as a repellent appears to be influenced primarily by their level of concern about being bitten by mosquitoes and by their concern that DEET may be harmful to health, despite its good safety record (49).

To prevent transmission of WNV through blood transfusion, blood donations in WNV-endemic areas should be screened by using nucleic acid amplification tests. Screening of organ donors for WNV infection has not been universally implemented because of concern about rejecting essential organs after false-positive screening results (50). Pregnant women should avoid exposure to mosquito bites to reduce the risk for intrauterine WNV transmission.

## Future Directions

WNV disease will likely continue to be a public health concern for the foreseeable future; the virus has become established in a broad range of ecologic settings and is transmitted by a relatively large number of mosquito species. WNV will also likely continue to spread into Central and South America, but the public health implications of this spread remain uncertain. Observations thus far in North America indicate that circulation of other flaviviruses, such as dengue, viral mutation, and differing ecologic conditions may yield different clinical manifestations and transmission dynamics. Over the next few years, research efforts might well be focused in several areas. Research into new methods to reduce human exposure to mosquitoes is crucial and can help prevent other mosquitoborne illnesses. This should include development of new methods to reduce mosquito abundance, development of new repellents, and behavioral research to enhance the use of existing effective repellents and other personal protective measures against mosquito bites. A better understanding of the dynamics of nonmosquitoborne transmission is essential to prevent disease among infants of infected mothers and recipients of blood transfusions and transplanted organs. Currently available prevention strategies such as the dissemination of knowledge and products for personal protection from mosquito exposure and the application of existing techniques for reducing mosquito abundance in communities at risk of WNV transmission need to be vigorously implemented. National and international surveillance for WNV transmission will be important to monitor spread of the virus and the effect of control strategies. Finally, further research into the ecologic determinants of WNV transmission, including climatic factors and dynamics of reservoir and vector populations, could help in determining geographic areas of higher risk for WNV disease.
